# Biomechanical Characterization of Endothelial Cells Exposed to Shear Stress Using Acoustic Force Spectroscopy

**DOI:** 10.3389/fbioe.2021.612151

**Published:** 2021-02-04

**Authors:** Giulia Silvani, Valentin Romanov, Charles D. Cox, Boris Martinac

**Affiliations:** ^1^Victor Chang Cardiac Research Institute, Sydney, NSW, Australia; ^2^St Vincent's Clinical School, University of New South Wales, Sydney, NSW, Australia

**Keywords:** cell mechanics, microrheology, endothelial cells, shear stress, junction, cytoskeleton, stress stiffening

## Abstract

Characterizing mechanical properties of cells is important for understanding many cellular processes, such as cell movement, shape, and growth, as well as adaptation to changing environments. In this study, we explore the mechanical properties of endothelial cells that form the biological barrier lining blood vessels, whose dysfunction leads to development of many cardiovascular disorders. Stiffness of living endothelial cells was determined by Acoustic Force Spectroscopy (AFS), by pull parallel multiple functionalized microspheres located at the cell-cell periphery. The unique configuration of the acoustic microfluidic channel allowed us to develop a long-term dynamic culture protocol exposing cells to laminar flow for up to 48 h, with shear stresses in the physiological range (i.e., 6 dyn/cm^2^). Two different Endothelial cells lines, Human Aortic Endothelial Cells (HAECs) and Human Umbilical Vein Endothelial Cells (HUVECs), were investigated to show the potential of this tool to capture the change in cellular mechanical properties during maturation of a confluent endothelial monolayer. Immunofluorescence microscopy was exploited to follow actin filament rearrangement and junction formation over time. For both cell types we found that the application of shear-stress promotes the typical phenotype of a mature endothelium expressing a linear pattern of VE-cadherin at the cell-cell border and actin filament rearrangement along the perimeter of Endothelial cells. A staircase-like sequence of increasing force steps, ranging from 186 pN to 3.5 nN, was then applied in a single measurement revealing the force-dependent apparent stiffness of the membrane cortex in the kPa range. We also found that beads attached to cells cultured under dynamic conditions were harder to displace than cells cultured under static conditions, showing a stiffer membrane cortex at cell periphery. All together these results demonstrate that the AFS can identify changes in cell mechanics based on force measurements of adherent cells under conditions mimicking their native microenvironment, thus revealing the shear stress dependence of the mechanical properties of neighboring endothelial cells.

## Introduction

Cells *in vivo*, whether isolated or part of a larger collective, are constantly exposed to physical forces such as extensional, compressive, and shear stresses, all playing a critical role in regulating physiological or pathological conditions (Huang et al., 2004; Le Roux et al., 2019). Cellular mechanics and rheological properties (e.g., viscosity and stiffness) determine the ability of cells to respond to mechanical cues, and are important for many physiological processes, including growth, division and migration (Hoffman and Crocker, 2009).

A remarkable example of cells under constant, potentially high mechanical stimuli are endothelial cells (ECs) that line the inner surface of blood vessels. ECs form a tight tessellated monolayer, i.e., the endothelium, which is constantly exposed to shear forces generated by blood flowing along their apical surface (Wang and Dimitrakopoulos, 2006a,b). The formation and maintenance of EC contacts, which provides the functional integrity of blood vessels, requires a complex interplay of plasma membrane proteins, cytoskeletal components and associated signaling molecules (Vestweber, 2000). As such, ECs have evolved to form a size-selective barrier, regulated by specialized transmembrane proteins, called inter-endothelial adherens junctions, located at the cell-cell border which are ubiquitously expressed in endothelia of all vascular beds (Petzelbauer et al., 2000). Among them, vascular endothelial cadherin (VE-cadherin), is one of the main structural and regulatory proteins controlling endothelial barrier function and permeability (Dejana et al., 2008; Vestweber, 2008; Giannotta et al., 2013). Not only do these junctions link the cells together, but they also work as surface receptors generating a cascade of intracellular signaling upon external applied stimuli (e.g., shear stress), triggering dynamic interactions with cytoskeletal elements. The shear stress exerted by blood flow, typically in the range of ~1–15 dyn/cm^2^ (Zarins et al., 1987; Sprague et al., 1997; Noria et al., 1999; Seebach et al., 2007; Tarbell, 2010; DeStefano et al., 2017), is known to be an important regulator of mechanotransduction in vascular physiology (Baratchi et al., 2017; Mehta et al., 2020), including mechanosensory processes of actin-mediated stabilization of junctions (Seebach et al., 2000, 2007; Schnittler et al., 2001; Helmke and Davies, 2002), followed by morphological remodeling (Vestweber, 2000; Ukropec et al., 2002; Tzima et al., 2005; Abu Taha and Schnittler, 2014) and tension redistribution (Conway and Schwartz, 2015) such that cells can maintain or change their shape.

According to the tensegrity model of living cells (Ingber, 1993), the shape and stability of neighboring cells are dictated by the internal framework of contractile actomyosin filaments, better known as actin stress fibers. These active filaments are interconnected with each other and with cell-cell or cell-extracellular matrix adhesion complexes, generating a tensile pre-stress through a balance of complementary forces within the network (Ingber, 1993; Ingber et al., 1994, 2014). Any variation of this structure, whether spatially or temporally, would affect the cell's mechanical properties, e.g., cell stiffness and deformability, as well as shape stability (Wendling et al., 1999; Wang et al., 2002; Canović et al., 2014; Gavara and Chadwick, 2016). Although the precise role of VE-cadherin as a sensor of external shear stress remains unclear, several studies have demonstrated the importance of shear stress in *in vitro* experiments to achieve a functional monolayer with barrier properties (Kim et al., 1989; Noria et al., 1999, 2004; Santaguida et al., 2006; Esch et al., 2011; Seebach et al., 2016; Gordon et al., 2020). Besides, it is widely accepted that any alteration of the blood flow pattern can lead to a wide range of vascular pathologies, including atherosclerosis and pulmonary arterial hypertension (Baeyens et al., 2016; Souilhol et al., 2020). As such, understanding how the mechanical behavior of collective ECs may vary when exposed to fluidic shear stress is of critical importance in elucidating cellular malfunction.

To date, the characterization of viscoelasticity of adherent cells has been addressed and investigated by several micro-rheological techniques including Atomic Force Microscopy (AFM), Magnetic or Optical tweezers, Micropipette Aspiration and Particle Tracking Microrheology (PTMR) (Martinac et al., 2020). Two major limitations of these techniques are measurement's reproducibility and throughput (Wu et al., 2018). More importantly, most of the techniques require an open chamber configuration, making it difficult to mimic the physiological shear stresses necessary for EC maturation during culture. An alternative method, the recently introduced Acoustic Force Spectroscopy (AFS) technology, has the potential to overcome these drawbacks and greatly improve investigation of adherent cell mechanics. Originally designed for single-molecule rheology, this method uses controlled acoustic forces (in the range of pNs to nNs) over a microfluidic channel to stretch multiple molecules in parallel that are individually tethered to functionalized microspheres (Sitters et al., 2015; Kamsma et al., 2016). Subsequently, it was used for characterization of the mechanical properties of red blood cells upon different chemical treatments (Sorkin et al., 2018). Recently, AFS methodology has been applied to Human Embryonic Kidney (HEK) cells capturing their inherent heterogeneity and showing the impact of temperature and pharmacological treatments on the mechanical properties at the membrane level (Romanov et al., 2020).

In this study, we exploit the unique configuration of the closed AFS channel to probe the modulation of mechanical properties of two different EC lines, i.e., Human Aortic Endothelial Cells (HAECs) and Human Umbilical Vein Endothelial Cells (HUVECs), during maturation of a confluent monolayer. By doing so, we build on the work of Nguyen et al. (2020), while overcoming the lack of physiologically relevant flow essential for development of a mature endothelial monolayer. First, we developed a protocol for long term, dynamic cell culture under physiological shear stress, i.e., 6 dyn/cm^2^. Second, after monolayer maturation, which was evaluated by immunofluorescence (IF) microscopy directly *in situ* at different steps, we performed creep tests, consisting of a staircase-like sequence of increasing force steps ranging through the physiologically relevant range of pNs–nNs, by locally pulling the periphery of the cellular membrane with functionalized silica particles. The viscoelastic response of the cells was then modeled by a Power law model, to estimate the two parameters characteristic of the model, i.e., stiffness and the power-law exponent. Under these conditions, we demonstrate the potential of the AFS as a tool for force measurements of adherent cells, under conditions mimicking their native microenvironment, also allowing for direct comparison between actin cytoskeleton reorganization, junction formation and shear stress induced stiffness modulation.

## Materials and Methods

### Experimental Set-Up

The AFS device consists of two glass layers hosting a microfluidic channel whose thickness and width are 100 μm and 2 mm, respectively. A transparent piezoelectric element glued on top allows the imaging of the sample in transillumination mode. The piezoelectric element was driven with a function generator in combination with an RF-amplifier to resonantly excite a planar acoustic standing wave over the chamber. A more detailed description of the AFS module developed by LUMICKS can be found in Kamsma et al. (2016). The resonant acoustic wave was exploited to exert forces over a range of pNs-nNs on hundreds of silica microspheres (diameter, 9.2 μm, Cospheric, Cat. No. SS05003) in parallel and with sub-millisecond response time. Data were acquired using the LUMICKS AFS technology including a LabVIEW interface dedicated for microsphere tracking with an integrated temperature controller (AFS-TC, LUMICKS).

### Fluid Dynamics Simulations

Simulations were performed using COMSOL Multiphysics 5.5. The Navier-Stokes equation was solved in 3D using the Microfluidic Module—Single Phase Flow–Laminar Flow sub-option. The no-slip boundary condition was used throughout. An inlet flow rate of 120 μL/min was used to obtain the shear forces reported here.

### Cell Culture and Seeding Into the AFS Channel

Human Umbilical Vein Endothelial cells (HUVECs) and Human Aortic Endothelial Cells (HAECs) were purchased from Lonza (Cat. No. CC-2517, Cat. No. CC-2535). The culture medium was the endothelial basal medium-2 (EBM-2) supplemented with endothelial growth medium (EGM-2) BulletKit from Lonza (Cat. No. CC-3162). Cells were grown in tissue culture flasks and maintained in humidified atmosphere at 37°C and 5% CO_2_. The culture medium was changed every 2 days and cells were used up to the 5th passage to ensure the expression of key endothelial protein components. The procedure to functionalize the microfluidic chip, adapted from Silvani et al. (2019), is here described in detail. Prior to cell seeding, the AFS chip was functionalized with fibronectin (100 μg/mL in EGM, Sigma-Aldrich, Cat. No F1141) using Tygon tubing (John Morris Scientific, Cat. NO ND-100-80), followed by incubation for 45 min at room temperature (RT). Culture flasks, with 80–90% cell confluence, were washed with Dulbecco Phosphate Buffered Saline (PBS) (Sigma Aldrich, Missouri, USA), detached using TrypLE™ solution (Gibco, Cat. No 12604-013) for 2 min at 37°C in 5% CO_2_ and blocked with growth media. Cell suspension was harvested and centrifuged at 1,400 rpm for 7 min and the supernatant was discarded. Cells were resuspended in EGM at an average concentration of 10^8^ cells ml^−1^ and transferred into a 1.5 ml Eppendorf tube. Using Tygon tubing, cells were then pulled through the channel up to the desired confluence of 60–70% and incubated at 37°C + 5% CO_2_ for 4 h, to let the cells attach to the bottom of the AFS channels under static conditions. After incubation, the endothelialized channel was ready either for acoustic measurements or to be connected to a peristaltic pump for growth media perfusion. Initially set to 66 μL/min, the flow rate was then increased to 120 μl/min (corresponding to a shear stress of 6 dyn/cm^2^) until completed 48 h of culture to achieve full junction maturation. All perfusion experiments were performed within a dry incubator to avoid damaging the AFS chip.

### Immunofluorescence Staining and Image Analysis

To investigate junction morphology and cytoskeleton organization under different experimental conditions, Immunofluorescence staining protocol was conducted directly in the acoustic chip on samples from different time points, i.e., after 4 h (static) or 48 h (flow culture). Fluorescence imaging of VE-cadherin protein and actin stress fibers was then performed and ImageJ software was used for analyzing the changes in morphology of both these proteins. Reagents were gently pulled into the AFS channel using tygon tubing and incubated under static conditions. Cells were first washed with 1 × PBS, fixed in 4% PFA for 15 min at room temperature, permeabilized for 2 min with Triton X-100 (0.5%, Sigma-Aldrich) and finally blocked with 1% BSA (Gibco, Invitrogen Corporation, Cat. No 30063-572). To monitor cell–cell contact, ECs were stained with VE-cadherin goat polyclonal primary antibody (200 mg/ml in 1% BSA) and incubated overnight. Cells were then incubated in the dark for 1 h at RT with a cocktail solution of AlexaFluor555 conjugate-Goat anti-Mouse IgG (H+L) Secondary Antibody (2 mg/ml, Invitrogen, Cat. No A21424) and Phalloidin (1:200, Sigma Aldrich, Cat. No P5282). Nuclei were stained with DAPI. Fluorescence images of the entire device were captured with an inverted microscope Nikon Eclipse Ti2-E, using Andor Zyla sCMOS camera in combination with a 20X objective (0.45 N.A.).

Changes in VE-cadherin and actin stress fibers organization were evaluated by performing line scans using ImageJ and analyzing the resulting fluorescence profile, as already done by Juffermans et al. (2009). For junction maturation at the cell-cell border, lines were drawn across the contact of two cells, and an average value of the maximum fluorescence intensity, over 50 measurements, were calculated for all experimental condition and cells types. A chart box is then plotted, showing the median maximum fluorescence intensity, scatter data points and error bars for each experimental conditions and cell lines.

For actin rearrangement, lines were drawn within individual cells, along the smaller axis, perpendicular to stress fibers. The procedure was repeated for 100 cells randomly chosen along the microfluidic channel. In particular, after image correction for background, the resulting fluorescence intensity profiles were filtered and analyzed for the number of peaks above an arbitrary baseline and at a defined distance from neighbors. In this way, two neighboring to*p*-values were considered as two separate peaks only when the distance between them was equal or higher than 1 μm. For each cell, the number of peaks was divided by the length of the scan line, resulting in the density of actin stress fibers (#/μm). A chart box is then plotted, showing the median density, scatter data points and error bars for each experimental conditions and cell lines.

### Microsphere Functionalization and Tracking

To let the cells interact with silica beads, the latter were functionalized with fibronectin (100 μg/mL in EGM) and incubated at RT on a rotating table for at least 1 h, as previously done (Grinnell and Geiger, 1986; Kessel et al., 2010). This coating screens the electrical charge on the surface of the bead when immersed in media (Nguyen et al., 2020). After incubation, the beads were injected into the endothelialized AFS channel at a cell to bead ratio of 1:2. The chip was placed on the AFS microscope stage for at least 5 min before being exposed to the acoustic force in order to allow for microsphere attachment to the cellular surface. At this stage of the experiment, the temperature controller was set to the physiological temperature of 37°C. Images were acquired with a bright-field inverted microscope equipped with a 1.3 MP camera recording at 60 Hz (UI-324CP, IDS) in combination with an air 20 × 0.75 NA objective [Nikon, CFI Plan APO, VC 20x (MRD70200)]. The bead z-position was determined using a predefined look-up-table (LUT) (Gosse and Croquette, 2002), a library of radial profiles as a function of z position with 100 nm steps, created from a series of microsphere images prior to the application of the acoustic force.

### Acoustic Force Calibration

To determine the acoustic radiation force, *F*_*rad*_, we performed a force-balance on acoustically driven beads. Briefly, when acoustic force is applied, beads are pushed toward the node of an acoustic standing wave. In such a scenario, the forces acting on a suspended bead in solution are the gravity force (*F*_*g*_), the buoyancy force (*F*_*b*_), the Stokes drag force (*F*_*D*_) and the acoustic radiation force (*F*_*rad*_). Assuming a constant velocity, all the forces at play cancel out:

(1) $$F_{g}-F_{b}+F_{D}-F_{rad}=0$$

(2) $$\frac{4}{3}\pi r^{3}\rho_{b}g-\frac{4}{3}\pi r^{3}\rho_{m}g+F_{D}=F_{rad}$$

where *g* is gravity, *r* is the bead radius, ρ_*b*_ is the silica beads density and ρ_*m*_ is the density of the cell media. While gravity and buoyancy forces are constant, the drag force *F*_*D*_ = *v*_*bead*_*γ*_*Brenner*_, acting on a bead moving normal to the surface, depends on bead velocity and was corrected for hydrodynamic surface effects using Brenner's factor (Cox and Brenner, 1961):

(3) $$\gamma_{Brenner}=\frac{6\pi \eta r}{1-\frac{9}{8}\left(\frac{r}{h}\right)+\frac{1}{\;2}{\left(\frac{r}{h}\right)}^{3}-\frac{57}{100}{\left(\frac{r}{h}\right)}^{4}+\frac{1}{15}{\left(\frac{r}{h}\right)}^{5}+\frac{7}{200}{\left(\frac{r}{h}\right)}^{11}}$$

where η is the media viscosity and h the height of the bead center to the surface (Schäffer et al., 2007).

The velocity was then calculated by recording the trajectory of beads moving toward the node and taking the derivative of the z-position over time. The bulk Stokes Drag coefficient, γ_0_ = 6πη*r*, was first inferred using Einstein relation and exploited to find the experimental medium viscosity, η, as described in Romanov et al. (2020), which was inserted in Equation (3) and used to calculate the effective drag coefficient. Finally, the radiation force, *F*_*rad*_, could be inferred from calculating the other forces. The procedure was repeated on multiple beads (*n* = 17) in EGM (η = 0.94E-3 Pa s), with increasingly applied voltage from 5 to 10 Vpp. The extrapolated forces were plotted against the applied voltage to demonstrate the quadratic dependence of the force with the amplitude Supplementary Figure 1.

### Force Steps Measurement

To measure the non-linear creep response of cells, a staircase-like pattern of increasing constant force (ranging from 186 pN to 3.3 nN), was applied for 10 s at each step, for a total of 80 s. We performed three independent experiments per conditions for both cell types. The corresponding stresses are calculated by dividing the force exerted on the bead, by the bead's surface (π*r*^2^) (ranging from 2.8 to 53.34 Pa). The displacement at each step was fitted by a creep-compliance model (see section below). All measurements were performed within 45 min at 37°C with a maximum peak-to-peak driving voltage of 45 V_pp_ at 14.50 MHz frequency.

### Data Fitting

To quantify the force dependence of the creep response we used a superposition of power-law models.

For each force step, we estimated the strain ϵ(t) as the bead displacement d(t) divided by the bead radius *r* and the stress σ as the applied force, *f*, divided by the bead cross-sectional area, *r*^2^π. The creep compliance *J*(*t*) could be determined using the expression $\frac{\text{d}\left(\text{t}\right)}{f}$ multiplied by a constant geometric factor *rπ* and fitted to the equation (Kollmannsberger et al., 2011):

(4) $$J\left(t\right)=\;J_{o}{\left(\frac{t}{\tau_{0}}\right)}^{\beta }$$

with time τ_0_ defined as 1. *J*_*o*_ is the softness and β is the power-law exponent, defining the solid- or liquid-like behavior of the cell. The value of β, typically falls in between 0.1 and 0.5 for most cells (Kollmannsberger and Fabry, 2011). The apparent elastic modulus, E_0_, the inverse of the creep prefactor *J*_*o*_, represents the stiffness and has a unit of Pa. The non-linear viscoelasticity (stress-stiffening behavior) was then revealed by plotting the elastic modulus, ${J_{o}\left(\sigma \right)}^{-1}$, *vs*. applied stresses.

### Statistics

Where applicable, the data sets to be analyzed were log-transformed and then compared based on methods described elsewhere (Ho et al., 2018). For completeness, statistical significance was tested by bootstrapping the sample 5,000 times and reporting the mean difference between the samples. The *p*-value is the likelihood of observing the effect size, if the null hypothesis of zero difference is true, based on a two-sided permutation *t*-test. For each permutation, 5000 reshuffles of the control and treatment groups were performed and compared. We report the back-transformed bootstrapped effect size and the corresponding 95% confidence interval. For example, an effect size of 2 means that the mean of sample 1 is 2-times larger than the mean of sample 2. Box and whisker plots display the median, 25% and 75% quartiles, with whiskers at an interquartile range of 1.5 (95% confidence). Outliers are shown as a red plus (+).

## Results

**Figure 1:**
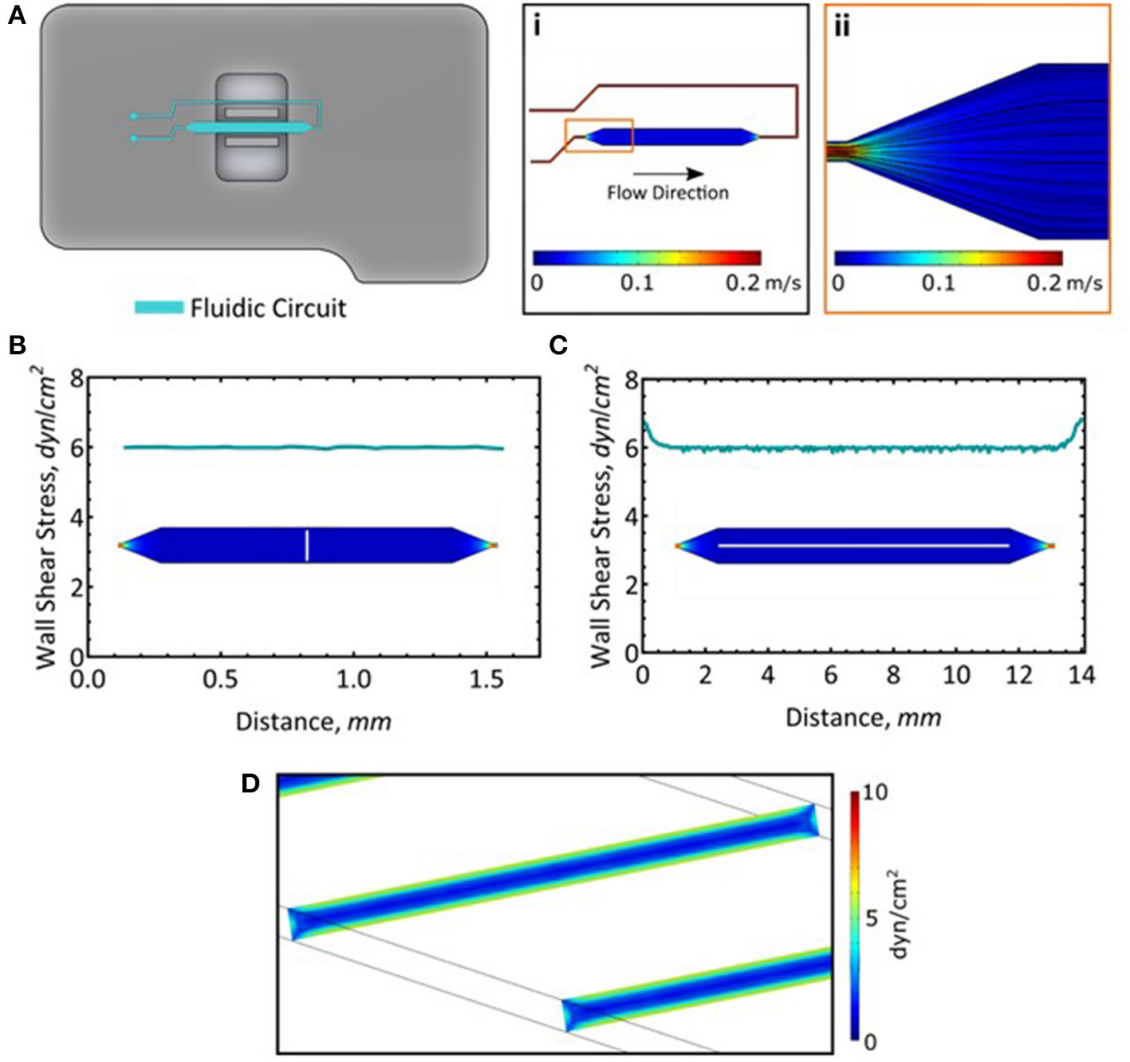
Long-term cell culture in the AFS chip. **(A)** Fluid dynamics simulation of flow and the resultant streamlines. **(B)** Wall shear stress measured across the channel. Measurement was taken along the white line. **(C)** Wall shear stress along the length of the channel. Measurement was taken along the white line. **(D)** A slice representation of the wall shear stress within the channel.

The main advantage of the AFS technology is the confined fluidic circuit, shown in Figure 1A. The device is also engineered and optimized to simultaneously allow for acoustic resonance and optical imaging thanks to a transparent piezoelectric element glued on top (Kamsma et al., 2016). The AFS is a two components system, one is the chip, and the other is the bead tracking software. The custom designed software (LUMICKS B.V.) can accurately track particle position, in real-time, in x, y, and z directions with accuracy between 5 and 20 nm (Kamsma et al., 2016, 2018; Sorkin et al., 2018).

Given the small dimension of the microfluidic channels (whose thickness and width are 100 μm and 2 mm, respectively), the flow rate and the properties of the fluid, the flow is fully laminar, meaning that its pattern is completely predictable and does not reach turbulence (Figure 1Ai,ii). Moreover, its solid structure allowed for high perfusion rates, mimicking the shear stress magnitude exerted on ECs monolayer *in vivo*. Here, we exploited the unique geometry of the AFS chip to perform long term culture of two different EC lines, i.e., HAECs and HUVECs, by connecting the microfluidic channel to a peristaltic pump and maintaining the flow of growth media for 48 h at a shear stress of 6 dyn/cm^2^. We confirmed the distribution of the shear stress across (Figure 1B) and along the length of the microfluidic channel (Figure 1C). The wall shear stress is steady and consistent at 6 dyn/cm^2^ (Figure 1D). It should be noted that while these simulations model the wall shear stress in the microfluidic channel, addition of a monolayer of cells will reduce the channel height by about 2 μm (Lambert et al., 2018) and as such, will experience most of the calculated maximal shear stress.

**Figure 2:**
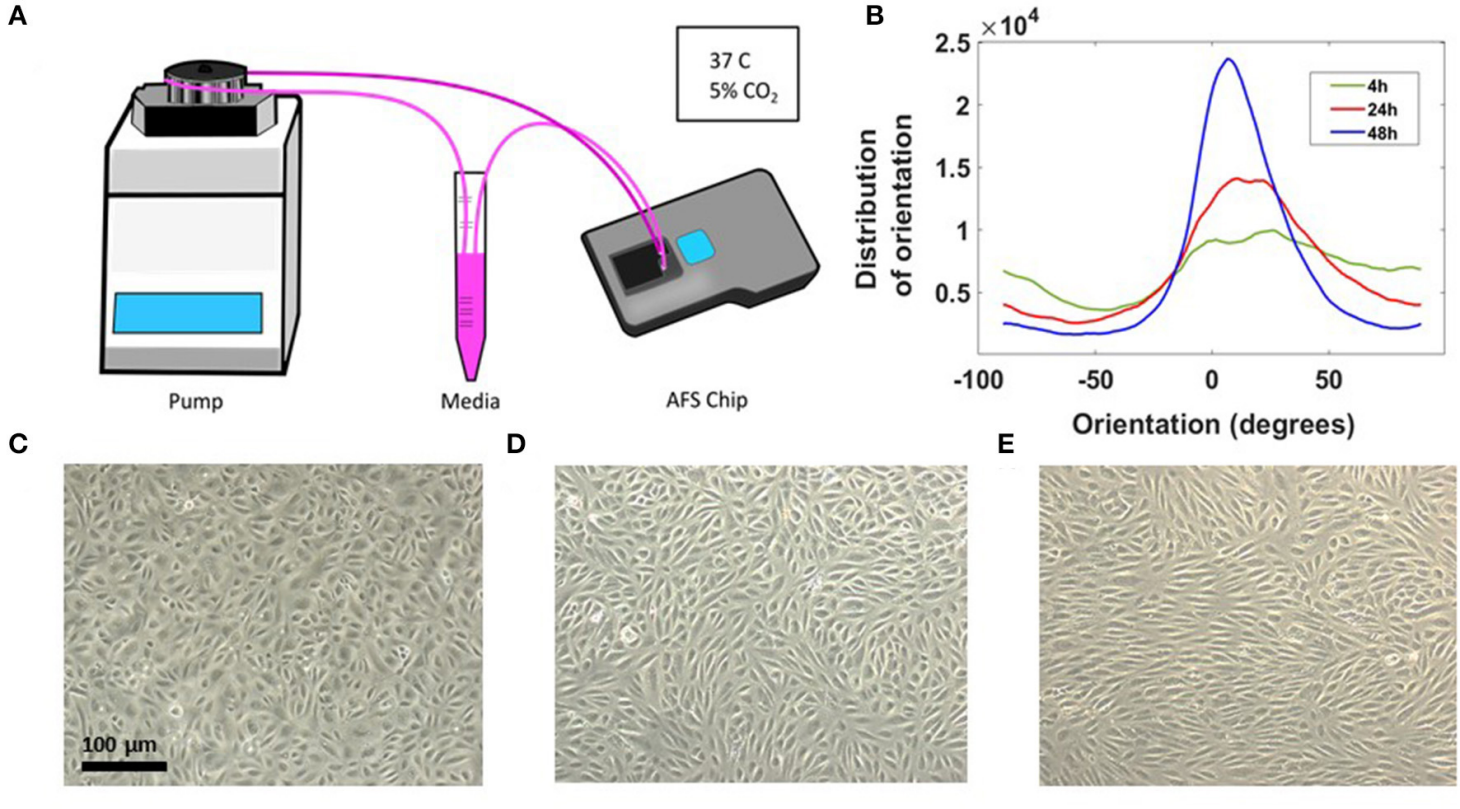
Long-term cell culture in the AFS chip. **(A)** Sketch of the set up with the AFS fluidic channel connected to a peristaltic pump. **(B)** Distribution of EC orientations after 4 h (green), 24h (red), and 48 h (blue) from seeding obtained with “orientationJ distribution” of ImageJ software. **(C)** Crop section of the channel imaged in transillumination mode, showing ECs 4 h from seeding procedure and **(D,E)** cells after 24 and 48 h, respectively, under flow culture condition at 37°C with 5% CO_2_ and no humidity. The flow was applied from left to right.

A sketch of the set-up is shown in Figure 2A. The chip and the medium reservoir were placed in a dry incubator at 37°C and 5% CO_2_ in order to avoid damaging the piezo-glass connection. During maturation, the endothelial morphology changed considerably, from an initial disorganized configuration of rounded spread cells to a compact endothelial monolayer, characterized by cell elongation in the stream direction (Figure 2B). Brightfield images of HAECs, after 4, 24, and 48 h from seeding procedure are shown in Figures 2C–E. The elongation of cells over time in the direction of the flow is indicative for a phenotypic endothelium (Lambert et al., 2018).

### Toward EC Monolayer and Junction Maturation

**Figure 3:**
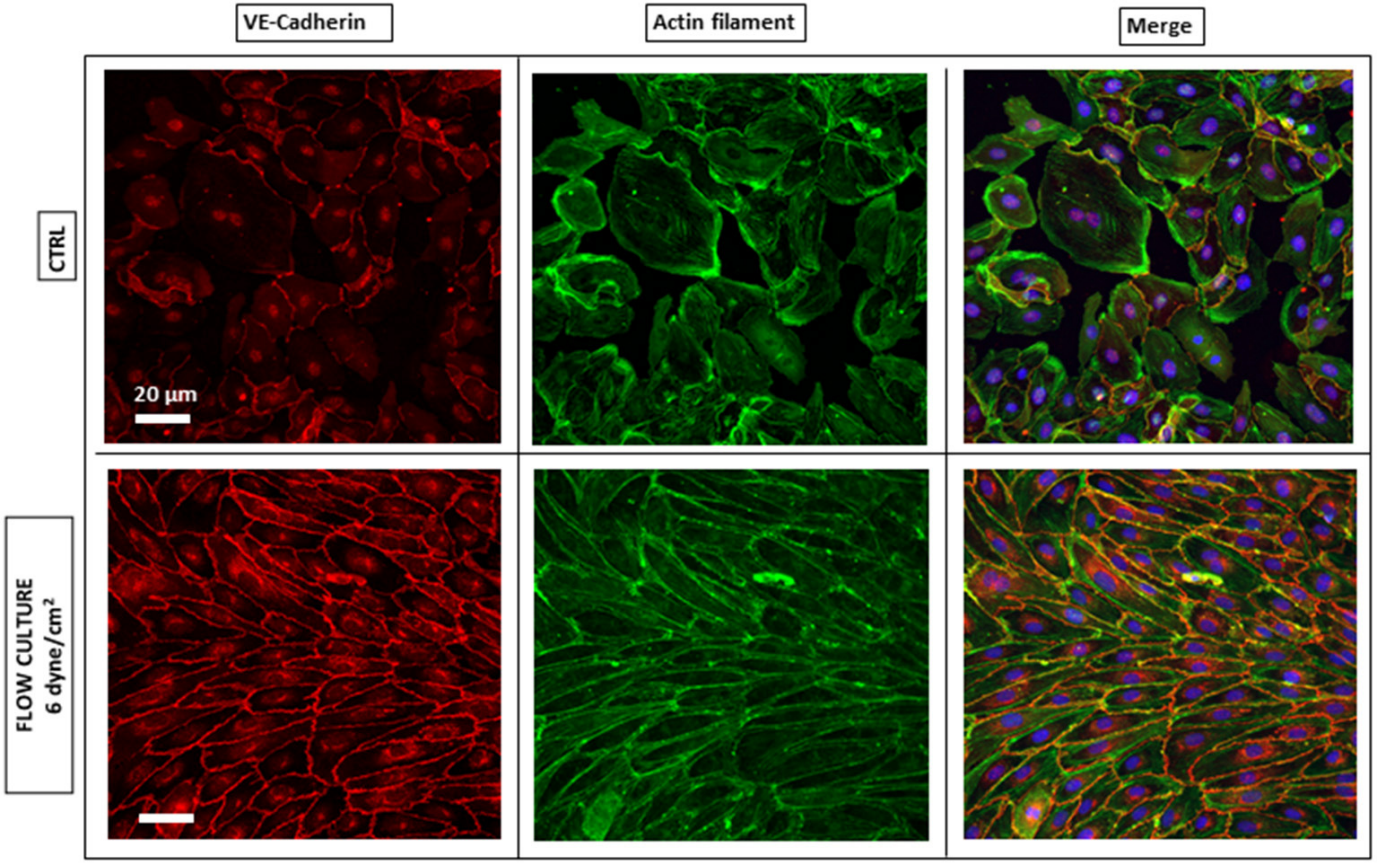
Endothelium maturation under flow culture condition. Fluorescence images of HAECs monolayer stained for junction VE-cadherin (red, left panel) and Actin Filaments (green, middle panel) under static (CTRL) or dynamic (Flow culture) condition.

It is generally proposed that the control of ECs adhesion, migration and barrier function, critically depends on the mutual interaction of VE-cadherin with actin filaments via receptor protein complexes (Drees et al., 2005; DuFort et al., 2011), as both structures are remodeled under physiological shear stress (Tzima et al., 2005; Abu Taha and Schnittler, 2014). Here, we performed IF microscopy to assess the phenotype of these key protein components, after 4 h from cells seeding into the AFS channel and at the end of the flow culture protocol (48 h), to demonstrate the significance of shear stress and timing in promoting cell barrier formation. In this section, we refer to HAECs staining and evaluation, although the same IF results were obtained with HUVECs (Supplementary Figure 2). When subjected to shear stresses, HAECs showed a varied response (Figure 3). The most striking one was the change in morphology, as they went from sub-confluence to complete confluence. Without any shear applied, i.e., CTRL, cells appeared larger and grew in a polygonal shape without directionality and with VE-cadherin proteins forming an irregular intermittent network (Figure 3, upper left panel) at the cell periphery. After 48 h of flow, cell contact was achieved and VE-cadherin exhibited linear oriented junctional staining (Figure 3, lower left panel) suggesting a well-established, mature confluent endothelium, as expected of a tight cobblestone monolayer (Jiménez et al., 2013).

**Figure 4:**
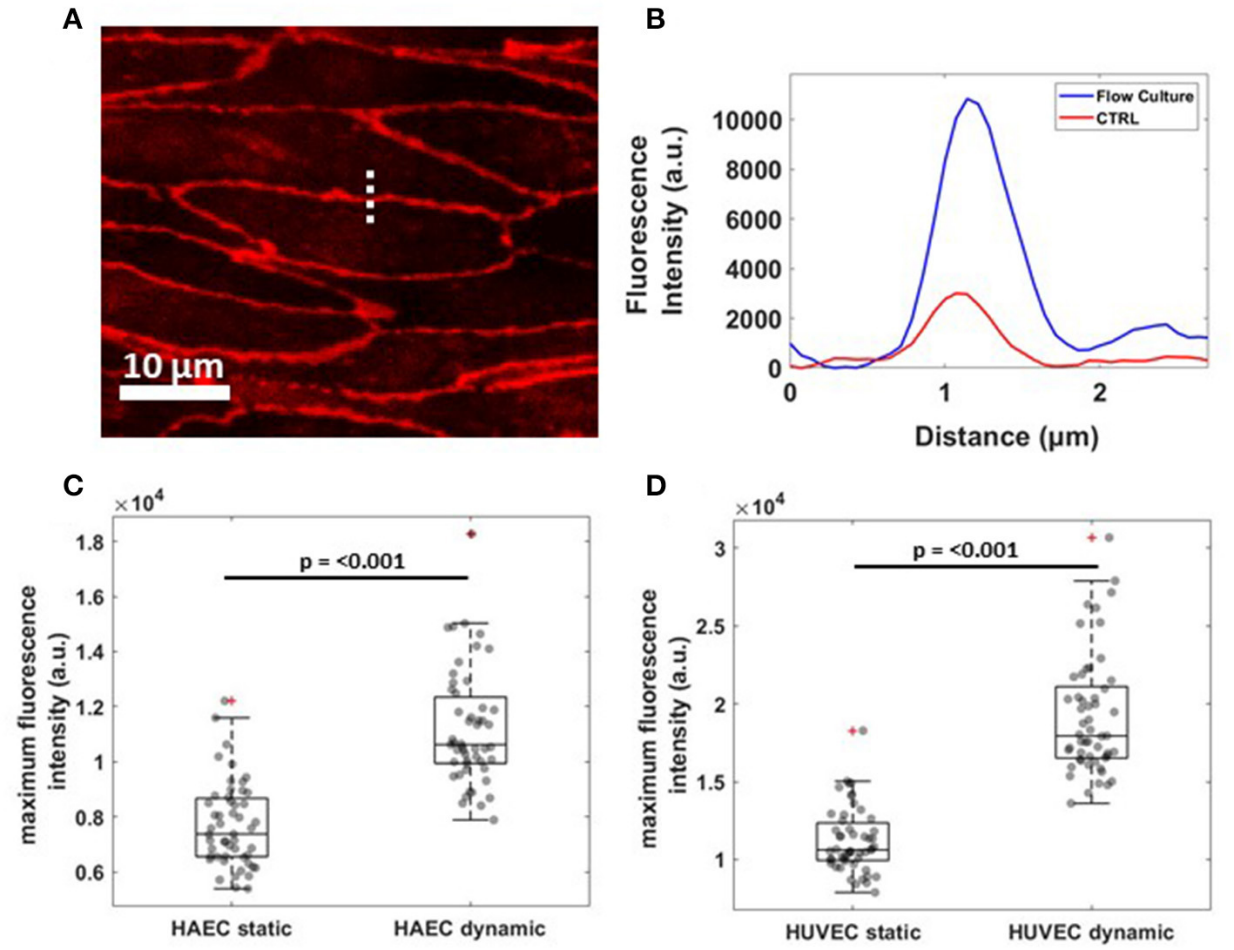
Reorganization of VE-cadherin under flow culture condition. **(A)** Dashed line across cell-cell junctions along which a fluorescence intensity profile was obtained. The image shows a crop of HAEC monolayer cultured under flow condition **(B)** Single representative graph showing fluorescence intensity (a.u.) profile for VE-cadherin signal under static (CTRL, red) and dynamic (Flow Culture, blue) condition. **(C,D)** Box plots and scatter data points (*n* = 50) for maximum fluorescence intensity analyzed for HAEC and HUVEC cells, respectively. *P*-value, effect size and confidence interval, for the comparison of dynamic vs. static condition are: *p* ≤ 0.001, ES = 1.45 [95% CI 1.36, 1.57] for HAEC and *p* ≤ 0.001, ES = 1.71 [95% CI 1.60, 1.83] for HUVEC.

To evaluate junction maturation at the cell-cell border, a fast and automated analysis method was performed on VE-cadherin fluorescence images by using a line scan across the contact of two ECs with ImageJ software and processing the resulting fluorescence profile in MATLAB software (Figure 4A). A single representative fluorescence intensity profile for VE-cadherin protein concentrated at the cell border for both experimental conditions is shown in Figure 4B, although the same fluorescence profiles have been founded for all the cells examined along the channel (data not shown). Under flow (blue curve), cells exhibited higher junctional localization of VE-cadherin staining, as opposed to those grown under static conditions (red curve), where the junction was yet to form. This result was found to be consistent for both cell lines with the median maximum fluorescence intensity, higher for cells exposed to the shear stress compare to the control (Figures 4C,D).

**Figure 5:**
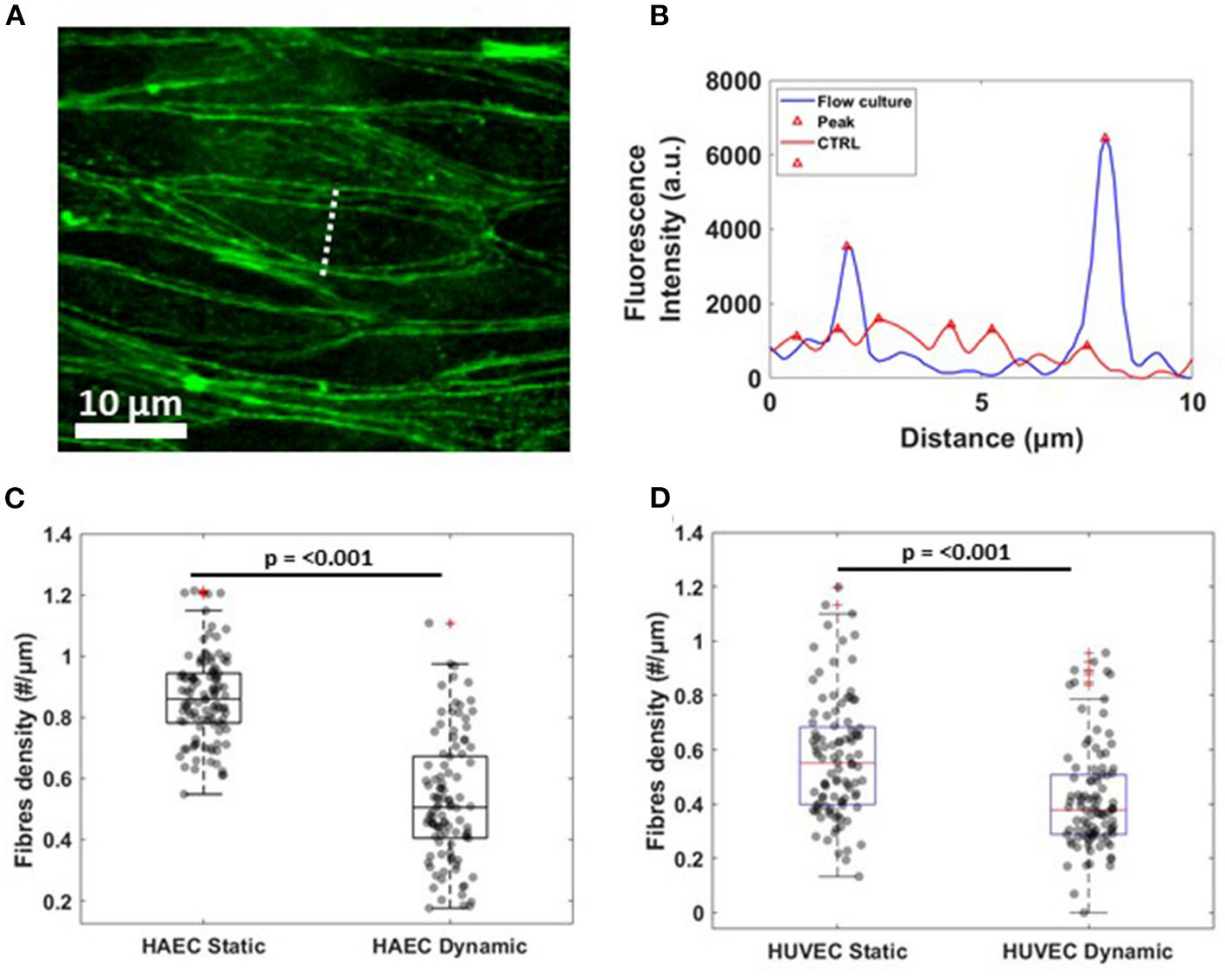
Actin fibers remodeling under flow culture condition **(A)** Dash line perpendicular to the width of a single cells along which fluorescence intensity profile was obtained. The image shows a crop of HAEC monolayer cultured under flow condition **(B)** Single representative graph showing fluorescence intensity (a.u.) profile for actin filament signal under static (CTRL, red) and dynamic (Flow Culture, blue) condition. **(C,D)** Box plots and scatter data points for F-actin stress fibers density analyzed for HAEC and HUVEC cells, respectively. *P*-value, effect size and confidence interval, for the comparison of dynamic vs. static condition are: *p* ≤ 0.001, ES = 1.44 [95% CI 1.2, 1.65] for HAEC and *p* ≤ 0.001, ES = 1.7 [95% CI 1.52, 1.87] for HUVEC.

Regarding the rearrangement of actin stress fibers within the cell's cytoplasm, Phalloidin staining revealed a strict cortical organization of actin filaments, referred to as circumferential actin bundles (Noda et al., 2010) in cells subjected to shear stress (Figure 3, lower middle panel), whereas cells under static conditions exhibited more stress fibers across the cell body (Figure 3, lower middle panel). Indeed, after dynamic culture, fluorescence imaging of the cytoskeleton in EC's showed actin filaments aligned along the cell periphery together with junction proteins, as shown in the lower right panel of Figure 3 by a linear overlayed pattern of VE-cadherin/Phalloidin. VE-cadherin actin stress fiber remodeling was also quantified using the same method with ImageJ software (Figure 5). Here, lines were drawn within individual cells, along the cells' smaller axis, perpendicular to stress fibers (Figure 5A) and the fluorescence intensity profile along the line revealed the number of fluorescence peaks, i.e., actin stress fibers (Figure 5B). Box and whiskers plot in Figures 5C,D show the median of fiber densities for each experimental conditions and cell lines, obtained over an average of 100 cells chosen randomly along the AFS channel. We found that, for both HUVECs and HAECs, the median density for cells under flow conditions decreased compared to control cells, suggesting that substantial remodeling of actin filaments is necessary for junction stabilization.

### Manipulating an Endothelial Monolayer With Acoustic Forces

**Figure 6:**
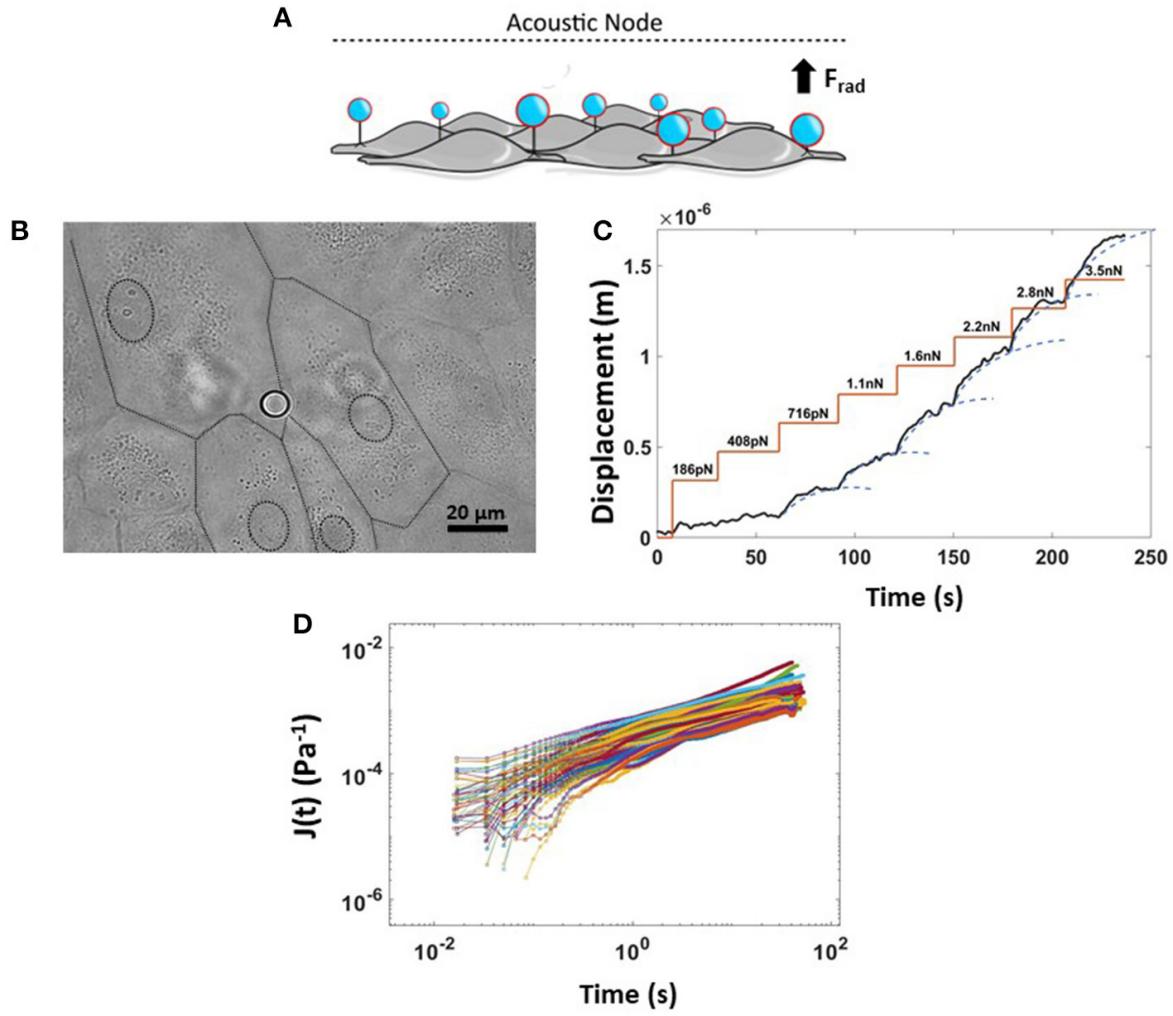
Rheology creep test for measuring non-linear viscoelastic properties of ECs. **(A)** Cartoon showing the physical principle of the experiment where application of an acoustic force leads to simultaneous displacement of many cell-attached beads. **(B)** Bright field image of endothelium monolayer seeded in the AFS channel together with Silica bead attached to the cellular surface. The dashed line represents the boundary of multiple cells. **(C)** Example of a single creep test made of a staircase-like sequence of increasing force steps, from 180 pN to 3.5 nN. The bead displacement is fit to a superposition of creep responses (blue dashed line). **(D)** Compliance curves taken from beads at a specific force step of 2.8 nN and collected into a single experiment over 45 min (*n* = 80, HAEC CTRL, EGM, 9.2 μm Silica beads). Data are presented as means ± SEM.

In our AFS experiments, the “endothelialized” microfluidic channel was resonantly excited at 14.50 MHz, the frequency of which determines the location of the nodal plane for the acoustic standing wave. When acoustic force is applied, pulling on silica microspheres attached to the cell surface toward the acoustic node stretches the membrane of multiple adherent cells, capturing the cell-dependent viscoelastic creep response. The principle of an AFS experiment is shown in Figure 6A. Thanks to the small diameter of silica beads (9.2 μm) compared to the cell's whole dimension, which is ~100 μm when subjected to shear stress, only beads in the near proximity of cell-cell borders were chosen for these experiments (Figure 6B). In order to study the dependence of endothelial mechanical properties with shear stress-mediated barrier functionality, a staircase-like sequence of increasing force steps, ranging from 186 pN to 3.5 nN, was applied in a single measurement to the endothelial monolayer (Figure 6C). Under this condition, multiple functionalized beads attached to cellular surfaces are pulled toward the acoustic node by an increasingly growing acoustic force, stretching the cellular membrane. Indeed, with the application of single stretch force or a single-step creep experiment, no reliable conclusion about force-dependent stiffness can be made, as the cytoskeletal structure may not return quickly enough to its original undisturbed state in between measurements, requiring a large number of independent measurements. Each step of the creep test was then fitted with a Power-Law model, $J\left(t\right)=\;J_{o}{\left(\frac{t}{\tau_{0}}\right)}^{\beta }$, to capture the viscoelastic parameters of cells, where E_0_, the inverse of the prefactor *J*_*o*_, is the apparent stiffness (units of Pascal) and β, the power-law exponent, defining the solid- or liquid-like behavior of the cell, typically in the range of 0.1–0.5 (Kollmannsberger and Fabry, 2011). The displacement of beads at each force step, always followed a weak power-law response (Figure 6C). Moreover, thanks to the field of view, using a 20X objective coupled with the system, tens of beads could be individually tracked, in real-time. We could typically collect about 80 compliance curves within 45 min, for a single experiment, as shown in Figure 6D.

**Figure 7:**
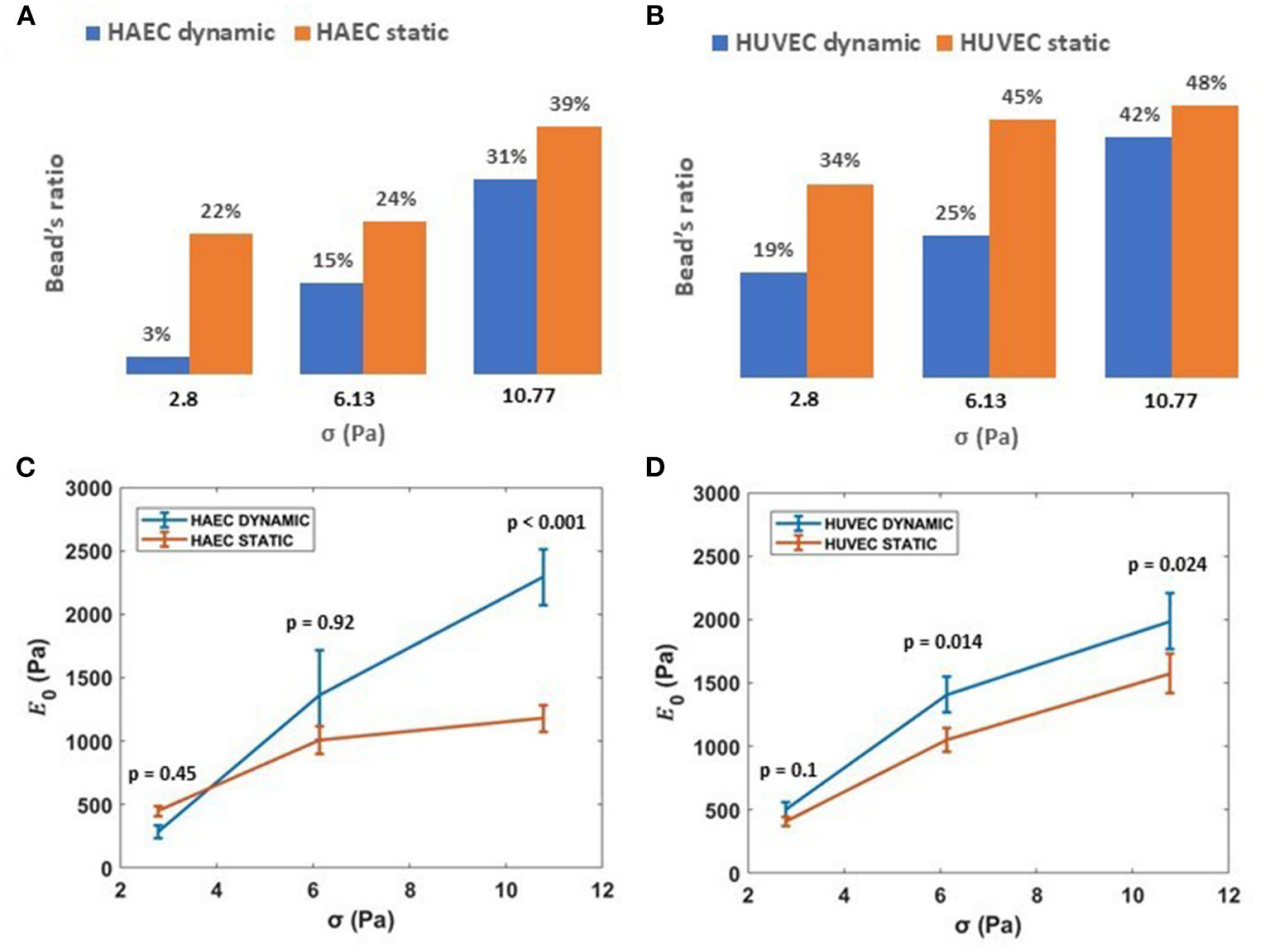
Effect of shear stress on ECs stiffness. **(A,B)** Ratio of beads displacing away from the cell when subject to applied acoustic pressure, grown under either static or dynamic conditions. **(C,D)** Stiffness, 1/J_0_, *vs*. applied external stress (i.e., 2.8, 6.13, 10.77 Pa) for HAECs and HUVECs, respectively, under static and dynamic condition. Data are presented as means ± SEM. *P*-value, effect size and confidence interval, for the comparison of dynamic vs. static condition at each force step are: *p* = 0.45, ES = 1.36 [95% CI 0.81, 1.83] for HAEC at 2.8 Pa, *p* = 0.92, ES = 1.02 [95% CI 0.65, 1.82] for HAEC at 6.13 Pa, *p* < 0.001, ES = 2.02 [95% CI 1.53, 2.67] for HAEC at 10.77 Pa and *p* = 0.1, ES = 1.4 [95% CI 0.98, 2] for HUVEC at 2.8 Pa, *p* = 0.014, ES = 1.54 [95% CI 1.1, 2.09] for HUVEC at 6.13 Pa, *p* = 0.024, ES = 1.40 [95% CI 1.07, 1.86] for HUVEC at 10.77 Pa.

Interestingly, the number of beads that result in membrane displacement was smaller for both HAECs and HUVECs under dynamic condition (Figures 7A,B). Cultured under 6 dyn/cm^2^, these cells are able to resist the deformation imposed by attached silica beads when subjected to increasing levels of stress than those cells cultured under static conditions. Nevertheless, cells under both experimental conditions, at increasing magnitudes of applied stresses (up to 10.77 Pa) show a stress-stiffening response, behaving like a viscoelastic material in the non-linear regime, where the stiffness, ${{E_{0}=J}_{o}}^{-1}$, increases with increasing stress (Figures 7C,D) in the order of 10^3^ Pa. This finding is in line with other rheology measurements of adherent cells using other methods (Mathur et al., 2000; Kataoka et al., 2002; Kollmannsberger et al., 2011; Marsh and Waugh, 2013; Vargas-Pinto et al., 2013; Wu et al., 2018). In particular, by choosing only beads in proximity to the cell-cell border, we found that the membrane cortex at cell periphery for both cell types appeared stiffer after 48 h of continuous physiological flow (Figures 7C,D, consistent with actin-mediated stabilization of junctions during monolayer maturation.

**Figure 8:**
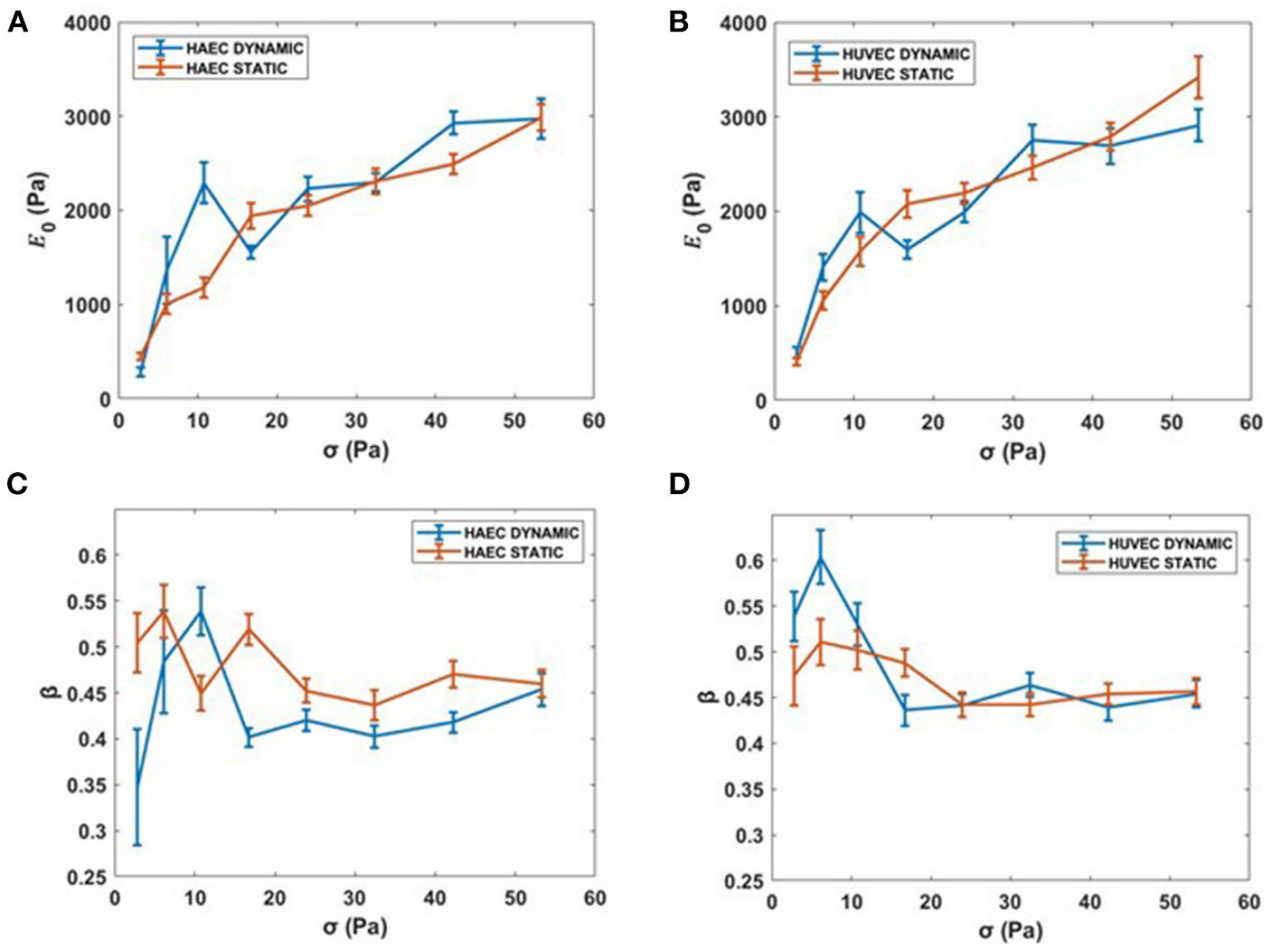
Complete creep response. **(A,B)** Stiffness *vs*. the complete applied external stress sequence (up to 53.34 Pa). **(C,D)** β vs. the complete applied external stress sequence. Data are presented as means ± SEM.

At higher forces, corresponding to stresses up to 53.34 Pa, cells still showed a stress-stiffening response, this rheological behavior has been found in the physiologically relevant regime of large external forces and deformation (Kollmannsberger et al., 2011). Although for cells subjected to the dynamic culture protocol, a drop of the apparent elastic modulus, E_0_, is observed at the specific force of 1.1 nN (i.e., 16.7 Pa) for both cell types (Figures 8A,B). Regarding the power law exponent, we found a heterogeneous behavior at lower stresses, which then stabilized to a value of 0.45 at the same stress applied of 16.7 Pa for both cell types (Figures 8C,D).

## Discussion

The mechanisms by which ECs respond and transduce signals, such as mechanical cues, are of great importance in vascular physiology, as they are often altered during disease, driving multifunctional behavior and pathology. It is broadly accepted that the cytoskeleton of adherent cells is critical to mechanotransduction processes by redistributing external force across the cytoplasm e.g., shear stress from the blood. Actin fibers are known to have a central role in force transmission, regulating many cellular functions, including morphological stability, adhesion, and motility (Wang et al., 1993; Chien, 2007). Although it is known that any changes in spatial distribution of actin fibers are shear dependent and occur in correlation with cadherin junction localization at cell borders (Hur et al., 2012), there is little quantitative knowledge about how stress fiber density and organization modulate cellular stiffness. Given that, it is important to characterize the mechanical properties of ECs under relevant physiological conditions in order to understand resulting cellular malfunction.

Much of the pioneering work characterizing adherent cells are performed using methods lacking in physiological reproduction of the cell's native microenvironment (Martinac et al., 2020). Experimental limitations, such as an open chamber configuration, make it difficult to assess EC monolayer maturation under physiological shear stress conditions during cell culture. Moreover, the thickness of the cell may alter indentation measurements as it will determine the contribution from the substrate to the overall stiffness (Dimitriadis et al., 2002; Rahimzadeh et al., 2011). Refer to Martinac et al. (2020) for a more comprehensive review on factors that may influence rheological measurements. In this study, we utilized a new technique, called Acoustic Force Spectroscopy, which operates in similar fashion to Optical Tweezers. AFS technology exploits acoustic mechanical waves to manipulate a particle's position and is composed of a driving element (piezo) and a recording element (software). The driving element is the oscillation of the piezo membrane that leads to the formation of acoustic waves that displace beads and the recording element is the custom designed tracking software capable of recording tens of beads, in real-time, at high resolution.

After establishing a mature endothelial monolayer, as assessed by immunofluorescence microscopy at different maturation steps, several beads at once were pushed toward the acoustic node, stretching different cellular membranes at the surface receptors/cytoskeleton linkage site in a high-throughput fashion. By fitting a power law model to the bead's displacement using a custom-written MATLAB program, we could determine the stiffness of the endothelial cells upon application of a range of forces and under different experimental conditions, revealing the shear stress dependence of the mechanical properties of neighboring endothelial cells. We showed that a relevant applied shear force leads to the re-arrangement of actin filament within the plasma membrane while simultaneously promoting junction formation and stabilization. Importantly, the shear stress experienced by ECs in our experiments was within the range of shear forces encountered by the cells in their native environment, allowing us to demonstrate how shear stress-driven morphology remodeling modulates cell stiffness.

The AFS was able to probe a change in membrane cortex's mechanical properties, which was found to be stiffer when stretched at the periphery of ECs after 48 h of continuous flow media. A surprising behavior of the plasma membrane was also captured, which was a sudden sharp drop in stiffness when pulled at a specific force of 1 nN. This behavior was found in both cell types and requires further future exploration as to its origin.

To further exploit the potential of the AFS, we performed immunofluorescent microscopy *in situ*, proving the applicability of the tool for future studies such as the investigation of actin filament remodeling during pulling experiments using live imaging techniques. Thanks to the micrometer dimension of silica beads, we also demonstrate the possibility to capture local mechanical properties selectively, by choosing beads in specific regions of interest. Next, it might be possible to choose different dimensions of silica beads, such as 5 μm in diameter, in order to discriminate the viscoelasticity of the cell's periphery from bulk membrane cortex and obtain a topographical map of cell's mechanical properties in high throughput.

In summary, our results demonstrate the potential of AFS as a novel tool for shedding light on mechanobiology of adherent cells cultured under physiologically relevant conditions. Further, we demonstrate that the AFS is capable of supporting fast and reproducible high-throughput single-cell measurements of viscoelasticity.

## Data Availability Statement

The raw data supporting the conclusions of this article will be made available by the authors, without undue reservation.

## Author Contributions

GS designed, ran, processed, analyzed the experiment, and wrote the manuscript. VR designed, ran, processed, analyzed the experiment, and edited the manuscript. CC helped with formulating experiments and editing of manuscript. BM formulated the experiments and editing the manuscript. All authors contributed to the article and approved the submitted version.

## Conflict of Interest

The authors declare that the research was conducted in the absence of any commercial or financial relationships that could be construed as a potential conflict of interest.

## References

[B1] Abu Taha A. Schnittler H. J. (2014). Dynamics between actin and the VE cadherin/catenin complex: novel aspects of the ARP2/3 complex in regulation of endothelial junctions. Cell Adh. Migr. 8, 125–135. 10.4161/cam.28243 24621569 PMC4049858

[B2] Baeyens N. Bandyopadhyay C. Coon B. G. Yun S. Schwartz M. A. (2016). Endothelial fluid shear stress sensing in vascular health and disease. J. Clin. Invest. 126, 821–828. 10.1172/JCI83083 26928035 PMC4767335

[B3] Baratchi S. Khoshmanesh K. Woodman O. L. Potocnik S. Peter K. McIntyre P. (2017). Molecular sensors of blood flow in endothelial cells. Trends Mol. Med. 23, 850–868. 10.1016/j.molmed.2017.07.007 28811171

[B4] Canović E. P. Seidl D. T. Barbone P. E. Smith M. L. Stamenović D. (2014). Stiffness versus prestress relationship at subcellular length scale. J. Biomech. 47, 3222–3225. 10.1016/j.jbiomech.2014.08.004 25138630 PMC4164390

[B5] Chien S. (2007). Mechanotransduction and endothelial cell homeostasis: the wisdom of the cell. Am. J. Physiol. Heart Circulat. Physiol. 292, H1209–H1224. 10.1152/ajpheart.01047.2006 17098825

[B6] Conway D. E. Schwartz M. A. (2015). Mechanotransduction of shear stress occurs through changes in VE-cadherin and PECAM-1 tension: implications for cell migration. Cell Adh. Migr. 9, 335–339. 10.4161/19336918.2014.968498 25482618 PMC4955370

[B7] Cox R. G. Brenner H. (1961). The slow motion of a sphere through a viscous fluid towards a plane surface. Chem. Eng. Sci. 16, 242–251. 10.1016/0009-2509(61)80035-3

[B8] Dejana E. Orsenigo F. Lampugnani M. G. (2008). The role of adherens junctions and VE-cadherin in the control of vascular permeability. J. Cell Sci. 121, 2115–2122. 10.1242/jcs.017897 18565824

[B9] DeStefano J. G. Xu Z. S. Williams A. J. Yimam N. Searson P. (2017). Effect of shear stress on iPSC-derived human brain microvascular endothelial cells (dhBMECs). Fluids Barri. CNS. 14, 1–15. 10.1186/s12987-017-0068-z 28774343 PMC5543552

[B10] Dimitriadis E. K. Horkay F. Maresca J. Kachar B. Chadwick R. S. (2002). Determination of elastic moduli of thin layers of soft material using the atomic force microscope. Biophys. J. 82, 2798–2810. 10.1016/S0006-3495(02)75620-8 11964265 PMC1302067

[B11] Drees F. Pokutta S. Yamada S. Nelson W. J. Weis W. I. (2005). α-catenin is a molecular switch that binds E-cadherin-β-catenin and regulates actin-filament assembly. Cell 123, 903–915. 10.1016/j.cell.2005.09.021 16325583 PMC3369825

[B12] DuFort C. C. Paszek M. J. Weaver V. M. (2011). Balancing forces: architectural control of mechanotransduction. Nat. Rev. Mol. Cell Biol. 12, 308–319. 10.1038/nrm3112 21508987 PMC3564968

[B13] Esch M. B. Post D. J. Shuler M. L. Stokol T. (2011). Characterization of *in vitro* endothelial linings grown within microfluidic channels. Tissue Eng. 17, 2965–2971. 10.1089/ten.tea.2010.0371 21895486 PMC3226056

[B14] Gavara N. Chadwick R. S. (2016). Relationship between cell stiffness and stress fiber amount, assessed by simultaneous atomic force microscopy and live-cell fluorescence imaging. Biomech. Model. Mechanobiol. 15, 511–523. 10.1007/s10237-015-0706-9 26206449 PMC4869747

[B15] Giannotta M. Trani M. Dejana E. (2013). VE-cadherin and endothelial adherens junctions: active guardians of vascular integrity. Dev. Cell. 26, 441–454. 10.1016/j.devcel.2013.08.020 24044891

[B16] Gordon E. Schimmel L. Frye M. (2020). The importance of mechanical forces for *in vitro* endothelial cell biology. Front. Physiol. 11:684. 10.3389/fphys.2020.00684 32625119 PMC7314997

[B17] Gosse C. Croquette V. (2002). Magnetic tweezers: micromanipulation and force measurement at the molecular level. Biophys. J. 82, 3314–3329. 10.1016/S0006-3495(02)75672-5 12023254 PMC1302119

[B18] Grinnell F. Geiger B. (1986). Interaction of fibronectin-coated beads with attached and spread fibroblasts: binding, phagocytosis, and cytoskeletal reorganization. Exp. Cell Res. 162, 449–461 10.1016/0014-4827(86)90349-6 3080317

[B19] Helmke B. P. Davies P. F. (2002). The cytoskeleton under external fluid mechanical forces: hemodynamic forces acting on the endothelium. Ann. Biomed. Eng. 30, 284–296. 10.1114/1.1467926 12051614

[B20] Ho J. Tumkaya T. Aryal S. Choi H. Claridge-Chang A. (2018). Moving beyond P values: everyday data analysis with estimation plots. bioRxiv Nat. Methods 26:2018 10.1101/377978 31217592

[B21] Hoffman B. D. Crocker J. C. (2009). Cell mechanics: dissecting the physical responses of cells to force. Annu. Rev. Biomed. Eng. 11, 259–288. 10.1146/annurev.bioeng.10.061807.160511 19400709

[B22] Huang H. Kamm R. D. Lee R. T. (2004). Cell mechanics and mechanotransduction: pathways, probes, and physiology. Am. J. Physiol. Cell Physiol. 287, C1–C11. 10.1152/ajpcell.00559.2003 15189819

[B23] Hur S. S. Del Alamo J. C. Park J. S. Li Y. S. Nguyen H. A. Teng D.. (2012). Roles of cell confluency and fluid shear in 3-dimensional intracellular forces in endothelial cells. Proc. Natl. Acad. Sic. U.S.A. 109, 11110–11115. 10.1073/pnas.1207326109 22665785 PMC3396533

[B24] Ingber D. E. (1993). Cellular tensegrity: defining new rules of biological design that govern the cytoskeleton. J. Cell Sci. 104, 613–627. 8314865 10.1242/jcs.104.3.613

[B25] Ingber D. E. Dike L. Hansen L. Karp S. Liley H. Maniotis A.. (1994). Cellular tensegrity: exploring how mechanical changes in the cytoskeleton regulate cell growth, migration, and tissue pattern during morphogenesis. Int. Rev. Cytol. 173–224. 10.1016/S0074-7696(08)61542-9 8169080

[B26] Ingber D. E. Wang N. Stamenović D. (2014). Tensegrity, cellular biophysics, and the mechanics of living systems. Rep. Progr. Phys. 77:046603. 10.1088/0034-4885/77/4/046603 24695087 PMC4112545

[B27] Jiménez N. Krouwer V. J. Post J. A. (2013). A new, rapid and reproducible method to obtain high quality endothelium *in vitro*. Cytotechnology 65, 1–14. 10.1007/s10616-012-9459-9 22573289 PMC3536875

[B28] Juffermans L. J. van Dijk A. Jongenelen C. A. Drukarch B. Reijerkerk A. de Vries H. E.. (2009). Ultrasound and microbubble-induced intra-and intercellular bioeffects in primary endothelial cells. Ultrasound Med. Biol. 35, 1917–1927. 10.1016/j.ultrasmedbio.2009.06.1091 19766381

[B29] Kamsma D. Bochet P. Oswald F. Alblas N. Goyard S. Wuite G. J.. (2018). Single-cell acoustic force spectroscopy: resolving kinetics and strength of T cell adhesion to fibronectin. Cell Rep. 24, 3008–3016. 10.1016/j.celrep.2018.08.034 30208324

[B30] Kamsma D. Creyghton R. Sitters G. Wuite G. J. Peterman E. J. (2016). Tuning the music: acoustic force spectroscopy (AFS) 2.0. Methods 105, 26–33. 10.1016/j.ymeth.2016.05.002 27163865

[B31] Kataoka N. Iwaki K. Hashimoto K. Mochizuki S. Ogasawara Y. Sato M. (2002). Measurements of endothelial cell-to-cell and cell-to-substrate gaps and micromechanical properties of endothelial cells during monocyte adhesion. Proc. Natl. Acad. Sic. U.S.A. 99, 15638–15643. 10.1073/pnas.242590799 PMC137769 12434019

[B32] Kessel S. Schmidt S. R. MÃller Wischerhoff E. Laschewsky A. Lutz J. F. O.. (2010). Thermoresponsive PEG-based polymer layers: surface characterization with AFM force measurements. Langmuir 26, 3462–3467. 10.1021/la903007v 19891449

[B33] Kim D. W. Gotlieb A. I. Langille B. L. (1989). In vivo modulation of endothelial F-actin microfilaments by experimental alterations in shear stress. Arteriosclerosis 9, 439–445. 10.1161/01.ATV.9.4.439 2751473

[B34] Kollmannsberger P. Fabry B. (2011). Linear and nonlinear rheology of living cells. Annu. Rev. Mater. Res. 41, 75–97. 10.1146/annurev-matsci-062910-100351

[B35] Kollmannsberger P. Mierke C. T. Fabry B. J. (2011). Nonlinear viscoelasticity of adherent cells is controlled by cytoskeletal tension. Soft Matter 7, 3127–3132. 10.1039/C0SM00833H

[B36] Lambert L. M. Pipinos I. I. Baxter B. T. Chatzizisis Y. S. Ryu S. J. Leighton R. I.. (2018). In vitro measurements of hemodynamic forces and their effects on endothelial cell mechanics at the sub-cellular level. Biomicrofluidics 12:064101. 10.1063/1.5028122 30473738 PMC6226388

[B37] Le Roux A. L. Quiroga X. Walani N. Arroyo M. Roca-Cusachs P. (2019). The plasma membrane as a mechanochemical transducer. Philos. Trans. R. Sock. B 374:20180221 10.1098/rstb.2018.0221 PMC6627014 31431176

[B38] Marsh G. Waugh R. E. (2013). Quantifying the mechanical properties of the endothelial glycocalyx with atomic force microscopy. JoVE 21:e50163. 10.3791/50163 23462566 PMC3605807

[B39] Martinac B. Nikolaev Y. A. Silvani G. Bavi N. Romanov V. Nakayama Y. (2020). Cell membrane mechanics and mechanosensory transduction. Curr. Topics Membr. Membr. Biomech. 86, 2–314. 10.1016/bs.ctm.2020.08.002 33837699

[B40] Mathur A. B. Truskey G. A. Reichert W. M. (2000). Atomic force and total internal reflection fluorescence microscopy for the study of force transmission in endothelial cells. Biophys. J. 78, 1725–1735. 10.1016/S0006-3495(00)76724-5 10733955 PMC1300769

[B41] Mehta V. Pang K. L. Rozbesky D. Nather K. Keen A. Lachowski D.. (2020). The guidance receptor plexin D1 is a mechanosensor in endothelial cells. Nature 578, 290–295. 10.1038/s41586-020-1979-4 32025034 PMC7025890

[B42] Nguyen A. Brandt M. Betz T. J. B. (2020). Microchip based microrheology via acoustic force spectroscopy shows that endothelial cell mechanics follows a fractional viscoelastic model. bioRxiv. 10.1101/2020.07.02.185330

[B43] Noda K. Zhang J. Fukuhara S. Kunimoto S. Yoshimura M. Mochizuki N. (2010). Vascular endothelial-cadherin stabilizes at cell–cell junctions by anchoring to circumferential actin bundles through α-and β-catenins in cyclic AMP-Epac-Rap1 signal-activated endothelial cells. Mol. Biol. Cell 21, 584–596. 10.1091/mbc.e09-07-0580 20032304 PMC2820423

[B44] Noria S. Cowan D. B. Gotlieb A. I. Langille B. L. (1999). Transient and steady-state effects of shear stress on endothelial cell adherens junctions. Circ. Res. 85, 504–514. 10.1161/01.RES.85.6.504 10488053

[B45] Noria S. Xu F. McCue S. Jones M. Gotlieb A. I. Langille B. L. (2004). Assembly and reorientation of stress fibers drives morphological changes to endothelial cells exposed to shear stress. Am. J. Pathol. 164, 1211–1223. 10.1016/S0002-9440(10)63209-9 15039210 PMC1615364

[B46] Petzelbauer P. Halama T. Gröger M. (2000). Endothelial adherens junctions, in Journal of Investigative Dermatology Symposium Proceedings (Amsterdam: Elsevier), 10–13. 10.1046/j.1087-0024.2000.00002.x 11147668

[B47] Rahimzadeh J. Meng F. Sachs F. Wang J. Verma D. Hua S. Z. (2011). Real-time observation of flow-induced cytoskeletal stress in living cells. Am. J. Physiol. Cell Physiol. 301, C646–C652. 10.1152/ajpcell.00099.2011 21653900 PMC3174563

[B48] Romanov V. Silvani G. Zhu H. Cox C. D. Martinac B. (2020). An acoustic platform for single-cell, high-throughput measurements of the viscoelastic properties of cells. Small. 10.1002/smll.202005759 33326190

[B49] Santaguida S. Janigro D. Hossain M. Oby E. Rapp E. Cucullo L. (2006). Side by side comparison between dynamic versus static models of blood–brain barrier in vitro: a permeability study. Brain Res. 1109, 1–13. 10.1016/j.brainres.2006.06.027 16857178

[B50] Schäffer E. Nørrelykke S. F. Howard J. (2007). Surface forces and drag coefficients of microspheres near a plane surface measured with optical tweezers. Langmuir 23, 3654–3665. 10.1021/la0622368 17326669

[B51] Schnittler H. J. Schneider S. W. Raifer H. Luo F. Dieterich P. Just I.. (2001). Role of actin filaments in endothelial cell-cell adhesion and membrane stability under fluid shear stress. Pflügers Archiv. 442, 675–687. 10.1007/s004240100589 11512023

[B52] Seebach J. Cao J. Schnittler H. J. (2016). Quantitative dynamics of VE-cadherin at endothelial cell junctions at a glance: basic requirements and current concepts. Discoveries 4:E63. 10.15190/d.2016.10 32309583 PMC7159836

[B53] Seebach J. Dieterich P. Luo F. Schillers H. Vestweber D. Oberleithner H.. (2000). Endothelial barrier function under laminar fluid shear stress. Lab. Invest. 80, 1819–1831. 10.1038/labinvest.3780193 11140695

[B54] Seebach J. Donnert G. Kronstein R. Werth S. Wojciak-Stothard B. Falzarano D.. (2007). Regulation of endothelial barrier function during flow-induced conversion to an arterial phenotype. Cardiovasc. Res. 75, 598–607. 10.1016/j.cardiores.2007.04.017 17531214

[B55] Silvani G. Scognamiglio C. Caprini D. Marino L. Chinappi M. Sinibaldi G.. (2019). Reversible cavitation-induced junctional opening in an artificial endothelial layer. Small 15:1905375. 10.1002/smll.201905375 31762158

[B56] Sitters G. Kamsma D. Thalhammer G. Ritsch-Marte M. Peterman E. J. Wuite G. J. (2015). Acoustic force spectroscopy. Nat. Methods 12, 47–50. 10.1038/nmeth.3183 25419961

[B57] Sorkin R. Bergamaschi G. Kamsma D. Brand G. Dekel E. Ofir-Birin Y.. (2018). Probing cellular mechanics with acoustic force spectroscopy. Mol. Biol. Cell 29, 2005–2011. 10.1091/mbc.E18-03-0154 29927358 PMC6232971

[B58] Souilhol C. Gauci I. Feng S. Tardajos Ayllon B. Mahmoud M. Canham L.. (2020). Homeobox B9 integrates bone morphogenic protein 4 with inflammation at atheroprone sites. Cardiovasc. Res. 116, 1300–1310. 10.1093/cvr/cvz235 31504243 PMC7243277

[B59] Sprague E. A. Luo J. Palmaz J. C. (1997). Human aortic endothelial cell migration onto stent surfaces under static and flow conditions. J. Vasc. Intervent. Radiol. 8, 83–92. 10.1016/S1051-0443(97)70521-9 9025045

[B60] Tarbell J. M. (2010). Shear stress and the endothelial transport barrier. Cardiovasc. Res. 87, 320–330. 10.1093/cvr/cvq146 20543206 PMC2915475

[B61] Tzima E. Irani-Tehrani M. Kiosses W. B. Dejana E. Schultz D. A. Engelhardt B.. (2005). A mechanosensory complex that mediates the endothelial cell response to fluid shear stress. Nature. 437, 426–431. 10.1038/nature03952 16163360

[B62] Ukropec J. A. Hollinger M. K. Woolkalis M. J. (2002). Regulation of VE-cadherin linkage to the cytoskeleton in endothelial cells exposed to fluid shear stress. Exp. Cell Res 273, 240–247. 10.1006/excr.2001.5453 11822879

[B63] Vargas-Pinto R. Gong H. Vahabikashi A. Johnson M. (2013). The effect of the endothelial cell cortex on atomic force microscopy measurements. Biophys. J. 105, 300–309. 10.1016/j.bpj.2013.05.034 23870251 PMC3714930

[B64] Vestweber D. (2000). Molecular mechanisms that control endothelial cell contacts. J. Pathol. 190, 281–291. 10.1002/(SICI)1096-9896(200002)190:3<281::AID-PATH527>3.0.CO;2-Z 10685062

[B65] Vestweber D. (2008). VE-cadherin: the major endothelial adhesion molecule controlling cellular junctions and blood vessel formation. Arterioscler. Thromb. Vasc. Biol. 28, 223–232. 10.1161/ATVBAHA.107.158014 18162609

[B66] Wang N. Butler J. P. Ingber D. E. (1993). Mechanotransduction across the cell surface and through the cytoskeleton. Science 260, 1124–1127. 10.1126/science.7684161 7684161

[B67] Wang N. Tolic-Nørrelykke I. M. Chen J. Mijailovich S. M. Butler J. P. Fredberg J. J.. (2002). Cell prestress. I. stiffness and prestress are closely associated in adherent contractile cells. Am. J. Physiol. Cell Physiol. 282, C606–C616. 10.1152/ajpcell.00269.2001 11832346

[B68] Wang Y. Dimitrakopoulos P. (2006a). Nature of the hemodynamic forces exerted on vascular endothelial cells or leukocytes adhering to the surface of blood vessels. Phys. Fluids 18:087107 10.1063/1.2336116

[B69] Wang Y. Dimitrakopoulos P. (2006b). Normal force exerted on vascular endothelial cells. Phys. Rev. Lett. 96:028106. 10.1103/PhysRevLett.96.028106 16486651

[B70] Wendling S. Oddou C. Isabey D. (1999). Stiffening response of a cellular tensegrity model. J. Theor. Biol. 196, 309–325. 10.1006/jtbi.1998.0841 10049624

[B71] Wu P. H. Aroush D. B. Asnacios A. Chen W. C. Dokukin M. E. Doss B. L.. (2018). A comparison of methods to assess cell mechanical properties. Nat. Methods 15, 491–498. 10.1038/s41592-018-0015-1 29915189 PMC6582221

[B72] Zarins C. K. Zatina M. A. Giddens D. P. Ku D. N. Glagov S. J. (1987). Shear stress regulation of artery lumen diameter in experimental atherogenesis. J. Vasc. Surg. 5, 413–420. 10.1016/0741-5214(87)90048-6 3509594

