# Supplementary material for: Biomechanical Characterization of Endothelial Cells Exposed to Shear Stress Using Acoustic Force Spectroscopy
Source: Front Bioeng Biotechnol. 2021 Feb 4;9:612151. doi: 10.3389/fbioe.2021.612151 (PMC7891662; doi:10.3389/fbioe.2021.612151)
Supplement: Supplementary file 1 [file Table_1.DOCX]

Supplementary Material

**Biomechanical characterization of endothelial cells exposed to shear stress using acoustic force spectroscopy**

**Giulia Silvani^1#^, Valentin Romanov^1#^, Charles D. Cox^1,2^ & Boris Martinac^1,2,*^**

^1^Victor Chang Cardiac Research Institute, Darlinghurst, Sydney, NSW, Australia

^2^ St Vincent’s Clinical School, University of New South Wales, Sydney, NSW, Australia

^#^These authors contributed equally

*** Correspondence:**
Corresponding Author
[b.martinac@victorchang.edu.au](mailto:b.martinac@victorchang.edu.au)

**
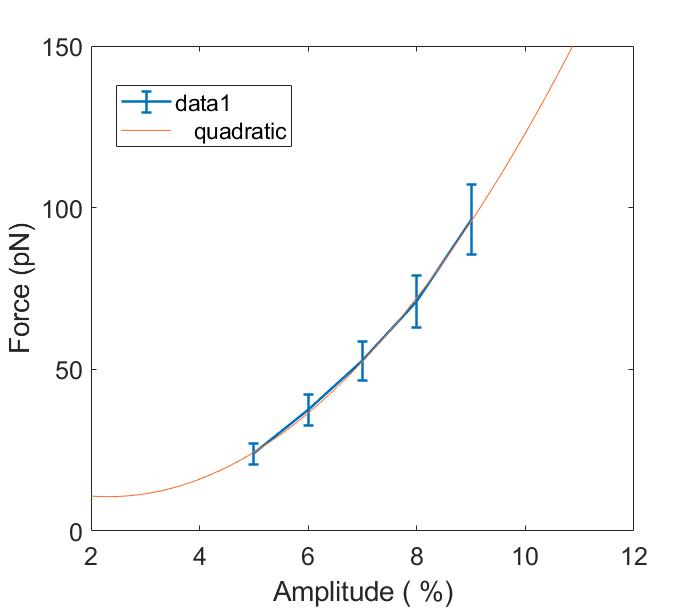
**

**Fig. S1.** Force calibration profile as a function of applied voltage (V_pp_) for the device used in these experiments (14.51 MHz, n=17, mean±SEM) and tested with 9.2 µm Silica particles in EGM (ν = 0.94E-3 Pa s).


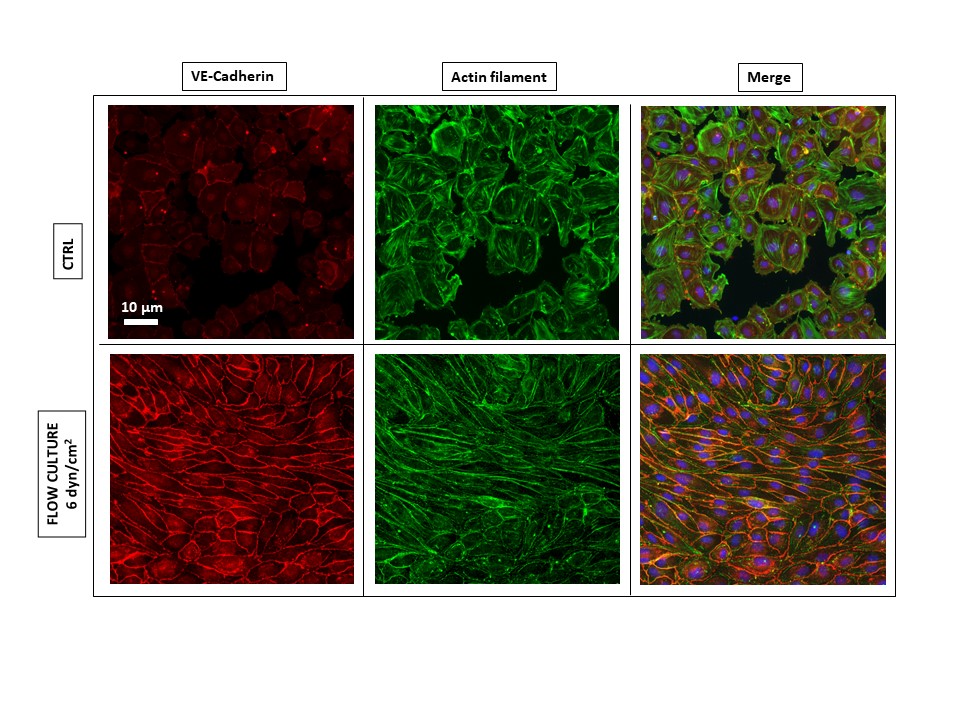


**Fig. S2.** Fluorescence images of HUVECs monolayer stained for junction VE-Cadherin (red, left panel) and Actin Filaments (green, middle panel) under static (CTRL) or dynamic (Flow culture) condition at 8 dyne/cm^2^.
